# Gayet-Wernicke’s encephalopathy complicating prolonged parenteral nutrition in patient treated for colonic cancer – a case report –

**DOI:** 10.1186/s40795-022-00585-w

**Published:** 2022-08-17

**Authors:** Skander Slim, Karim Ayed, Wissem Triki, Abdelmajid Baccar, Oussama Baraket, Khaled Rahal, Imen Ganzoui, Sami Bouchoucha

**Affiliations:** 1 Surgical Oncology Department, Salah Azaiez Institute of Cancer, Tunis, Tunisia; 2 Surgical Department, Habib Bougatfa Hospital, Bizerta, Tunisia; 3 Radiology Department, Habib Bougatfa Hospital, Bizerta, Tunisia

**Keywords:** Gayet-Wernicke encephalopathy, Thiamine, Vitamin B1, Parenteral nutrition, Case report

## Abstract

**Background:**

Gayet-Wernicke’s encephalopathy (GWE) is a neurological pathology caused by a Thiamine deficiency. While it is most often related to chronic alcoholism, GWE can occur in any situation that results in thiamine deficiency. It is a fairly common pathology that is frequently underdiagnosed and therefore under-treated, and is associated with a high mortality and morbidity rate. In the absence of pathognomonic signs, the diagnosis of GWE relies on a range of clinical, biological and radiological assessments. GWE is considered a medical emergency.

We present a case of Gayet-Wernicke’s Encephalopathy resulting from complete parenteral nutrition in an undernourished North African male operated for a left colon tumor. Through this report, our aim was to put the light on this often underknown disease and to remind the interest of thinking about this pathology in patients at risk of undernourishment especially in oncology.

**Case presentation:**

A 66-year-old North African male with no personal or family history was operated for a sigmoid colon tumor. He was put on exclusive parenteral nutrition on day thirteen post-operatively and presented with a GWE on day sixteen post-operatively. The patient was treated with intravenous vitamin B1 on day eighteen post-operatively and deceased on day twenty-four post-operatively.

**Conclusions:**

Although most often associated with chronic alcoholism, GWE occurs in any situation where there is an increased energy demand or decreased nutritional intake especially in oncology.

GWE is common but under-diagnosed and remains lethal if not treated urgently, hence the importance of prophylactic treatment.

## Background

Gayet-Wernicke’s encephalopathy (GWE) is a potentially lethal neurological emergency caused by a thiamine deficiency. Although it is most often associated with chronic alcoholism, it can also occur in any situation that results in thiamine deficiency.

Underdiagnosed despite its frequency, GWE has a poor prognosis with high morbidity and mortality. The absence of pathognomonic signs makes it difficult to diagnose, and relies on a range of clinical, biological and radiological assessments.

This pathology is treatable, whereby several studies have proposed Thiamine treatment regimens, both prophylactic and curative, with promising results.

We report a case of Gayet Wernicke’s encephalopathy caused by complete parenteral nutrition in an undernourished patient operated for a left colon tumor.

## Case presentation

A 66-year-old North African male with no past medical or family history consulted for the management of a left iliac fossa pain evolving over the course of two months. He was retired and did no smoke nor consume alcohol. He was not undergoing any medical treatment. The patient’s chief complaints included an alternation of diarrhea and constipation, as well as asthenia.

On admission, it was found that the patient was underweight (BMI = 17) with an average general condition (PS = 2) and pale conjunctivae.

Physical examination found no fever (Temperature = 37,3 °C), normal blood pressure (120/80 mmHg) and normal pulse (80 bpm). Neurological examination was normal (GCS = 15/15).

Abdominal examination found a tenderness in the left iliac fossa with no obvious palpable mass. There was no evidence of hepatomegaly nor splenomegaly.

The lymph node areas were free. The digital rectal exam was normal, with no rectal bleeding and no identifiable intra-rectal mass.

The biological assessment showed:

- Blood Group: B ( +) positive
- CBC: WBC = 10,40 × 103/ μL, #Neu = 4,82 × 103/ μL, #Lym = 3,18 × 103/ μL, #Mono = 0,72 × 103/ μL, #Eos = 0,29 × 103/ μL, #Baso = 0,05 × 103/ μL, RBC = 3,57 × 106/ μL, Hb = 12 g/dL, HCT = 33%, MCV = 84 fL, MCH = 28,9 pg, MCHC = 31,8 g/dL, RDW = 14,2%, Platelet = 235 × 103/ μL, MPV = 9,5 fL, Na^+^ = 146 mmol/L, K^+^ = 3,8 mmol/L, Urea = 5,4 mmol/L, Creatinine = 70 μmol/L, Alanine aminotransferase = 45 IU/L, Aspartate aminotransferase = 43 IU/L, Gamma-Glutamyl Transferase = 70 IU/L, Alkaline Phosphatase = 93 IU/L, Total bilirubin = 17 μmol/L, Conjugated bilirubin = 5 μmol/L, Albumin = 55 g/L.

The patient had an abdominopelvic computed tomography (CT) scan which showed a 12 cm long axis, stenosing sigmoid tissue mass associated with left pelvic adenopathy of 1,7 cm.

A colonoscopy showed an ulceroburging process of the sigmoid, the biopsy of which revealed a moderately differentiated Lieberkuhnian adenocarcinoma. The locoregional and distance extension assessments were negative. The patient underwent surgery.

Per-operatively, a 20 cm long axis invading tumor was found at the sigmoid colon complicated with a peri-neoplastic abscess. However, several adhesions did not allow exploration of the liver. There was neither ascites liquid nor carcinosis nodules.

The patient had a monobloc resection of the sigmoid colon and invaded hail with end-to-end hail-to-hail anastomosis with the confection of a double Bouilly Walkman stoma.

During the first post-operative week, the patient was on alternate infusions of saline and glucose serum. On day seven post-operatively, we introduced oral feeding, which was not well tolerated by the patient.

The postoperative period was marked by installing soft white edema of the lower limbs keeping the cup associated with ionic disorders such as hyponatremia (Na^+^ = 130 mmol/L) and hypokalemia (K^+^ = 2,9 mmol/L) associated with hypo-protidemia (Protein = 44 g/L) and hypo-albuminemia (Albumin = 26 g/L). The patient was fully conscious Neither tachycardia nor arterial hypotension were noted.

After regulating ionic disorders, and given the patient’s undernourished condition, he was placed on exclusive parenteral nutrition on day thirteen post-operatively. On day sixteen post-operatively, the patient presented a sudden worsening of his neurological state with a fluctuating GCS between 10–11/15 without convulsions. Physical examination found no fever and no infectious signs (clear urine, no veinitis, clean surgical wound).

The biological report found no metabolic disorders.

The patient’s altered neurological status was accompanied by a drop in blood pressure and worsening of edema in the lower limbs which required sedation, intubation, and vasoactive drugs.

A cerebral and thoracic-abdominal-pelvic CT scan were performed with no abnormalities.

**Fig. 1:**
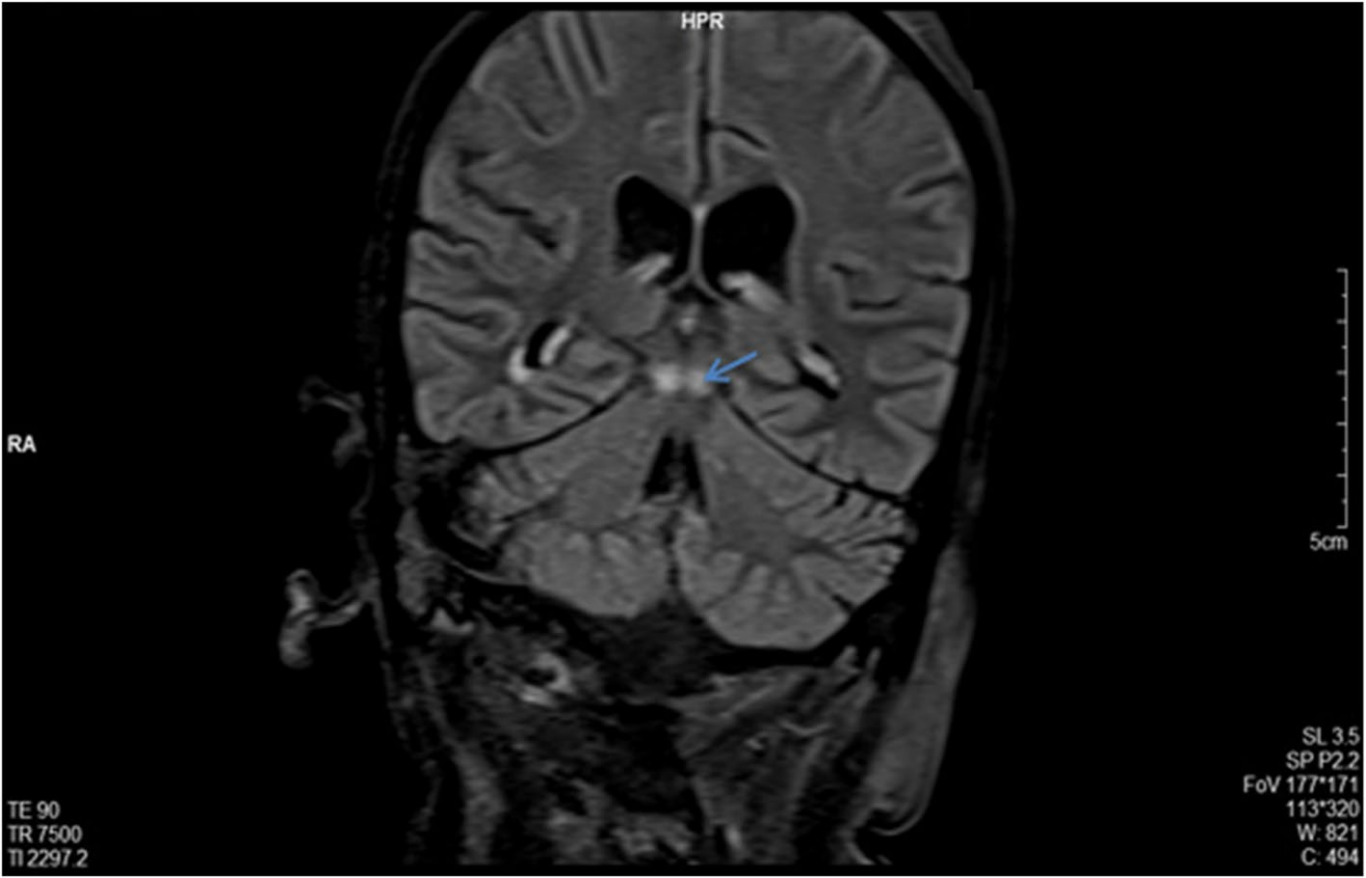
Coronal section of Cerebral MRI in FLAIR sequence showing: Hyper signal of the mammary bodies

**Fig. 2:**
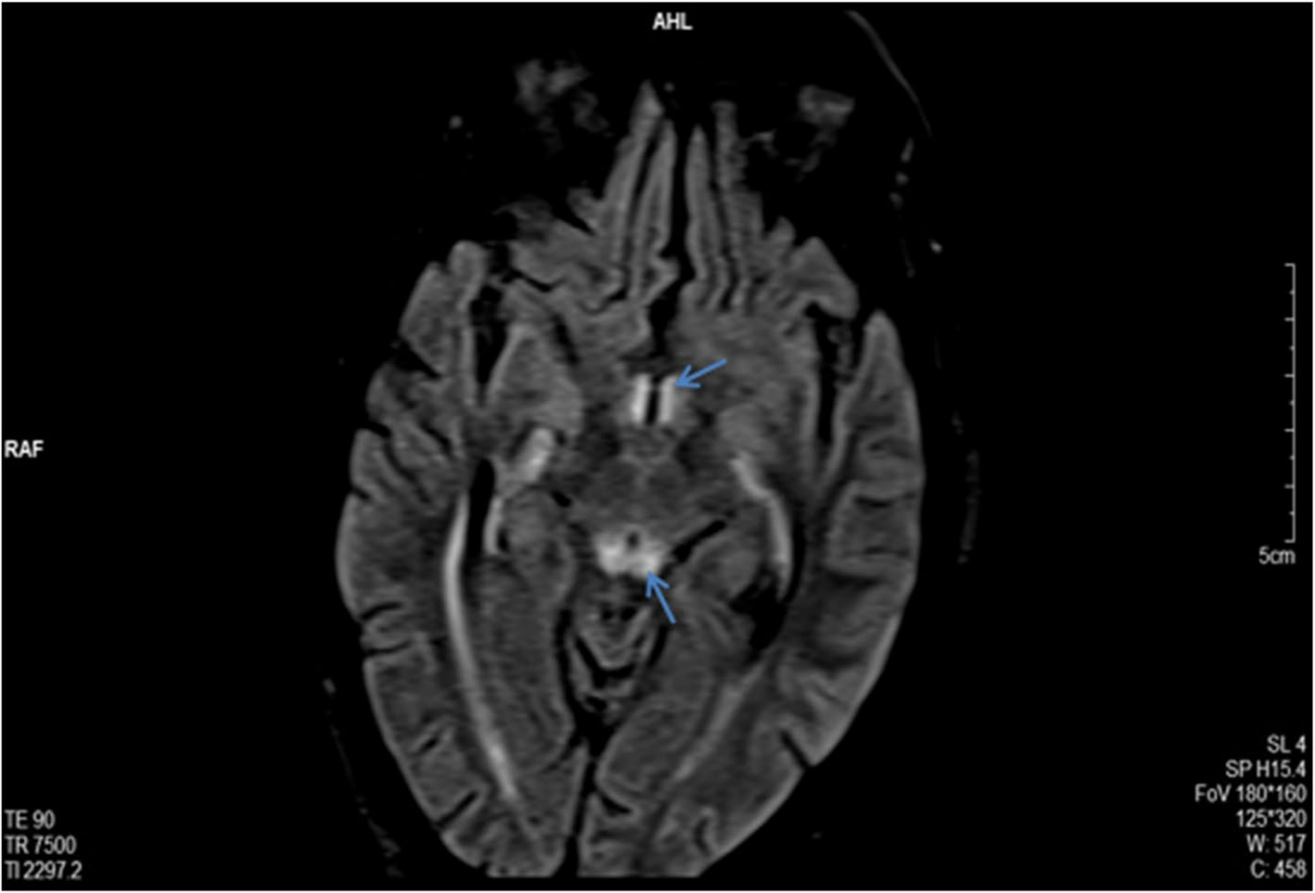
Axial section of Cerebral MRI in FLAIR sequence showing: Hyper signal of the mammary and peri-aqueductal bodies

The patient had a cerebral magnetic resonance imaging (MRI), which showed a hyper signal of the mammary bodies (in FLAIR coronal section) (Fig. 1) and a hyper signal of the mammary and peri-aqueductal bodies (in FLAIR axial section) (Fig. 2).

This aspect was compatible with Gayet-Wernicke’s encephalopathy and a right anterior frontal juxta-ventricular lesion measuring 1,5 cm long axis, corresponding to a cavernoma or a secondary hemorrhagic lesion.

The patient underwent vitamin B1 1000 mg/day intravenous (high dose) for three days starting on day eighteen post-operatively.

On day twenty-four post-operatively, even under high doses of vasoactive drugs, patient was 80/50 blood pressure and desaturated at 92% SpO2. He then experienced a cardiorespiratory arrest which did not respond to adequate resuscitation. No precipitating condition for the cardiorespiratory arrest could be formally identified, but high output failure remains the most likely cause.

## Discussion

After observations made by James Jackson (1822) and Charles Gayet (1875), Gayet-Wernicke’s encephalopathy was first described by Carl Wernicke in 1881 [1].

It is a severe deficiency encephalopathy linked to a vitamin B1 insufficiency. Epidemiological data is variable, and its global prevalence is estimated to vary between 0 and 2.8%, depending on the region [2]. It is relatively high in cases of parenteral nutrition without vitamin supplementation [3, 4].

S. Lescuyer and al estimate that Gayet Wernicke’s Encephalopathy is under-diagnosed, with a clinical prevalence of 0.04% to 0.13% compared to 0.8% and 2.8% in pathology [5].

Initially reported in malnourished alcoholic patients, this condition can be seen in any situation causing severe undernutrition, including hyperemesis gravidarum, thyrotoxicosis, peptic ulcer and stomach cancer, duodenal or jejunal resection, bariatric surgery, anorexia nervosa, prolonged fasting, and prolonged parenteral nutrition [5–9].

Dietary thiamine acts as a cofactor of several enzymes involved in the Krebs cycle and the phosphate pentose cycle [9]. Their deficiency is the cause of focal lactic acidosis due to insufficient brain energy, leading to an alteration of the blood–brain barrier and cell death by necrosis and apoptosis [10]. For adults, the daily requirement of Thiamine is 1.2 mg/day for men and 1.1 mg/day for women and its biological half-life in the body is 9 to 18 days, with a low total pool of about 30 mg [10]. It explains the rapid depletion of reserves in case of undernutrition [11]. Our patient’s onset of symptoms was sixteen days.

Clinically, it is manifested by the classic triad: confusional syndrome, ataxia, and oculomotor disorders, which is present in only 16% to 38% of cases, hence the interest of thinking about this diagnosis in any undernourished patient presenting neurological disorders of sudden onset [12].

In addition to these neurological manifestations, thiamine deficiency can lead to a picture of high output heart failure that is often misunderstood and mistaken for coronary ischemia or septic shock [11]. The blood thiamine level may be normal despite clinical signs of insufficiency, requiring the use of specific tests (thiamine diphosphate and red blood cell thiamine transketolase activity) if there is diagnostic doubt [12]. Some authors also report that hypomagnesaemia may be a cause of the occasional thiamine refractoriness and correction of hypomagnesaemia is accompanied by the recovery of blood transketolase activity and total clearing of oculomotor disorders [13].

Magnetic resonance imaging (MRI) plays an essential role in the diagnosis of Gayet-Wernicke’s encephalopathy. It typically shows hyper signals in T2, FLAIR, and diffusion sequences in the periaqueductal (Fig. 2), around the third ventricle, the medial thalamus, and the mammary bodies (Fig. 1). Although very evocative, these signal anomalies are not pathognomonic (93% specificity). The primary differential diagnoses must therefore be eliminated: cerebrovascular accident, cerebral venous thrombosis, acute encephalitis, and cerebral lymphoma. It should also be noted that MRI did not show any anomalies in 53% of cases [11]. Therefore, normal imaging should not exclude the diagnosis [11, 14].

Gayet-Wernicke’s encephalopathy is a therapeutic emergency. Intravenous thiamine should be administered as soon as the diagnosis is made [11].

The widely adopted treatment regimen is that of the National Institute of Health and Clinical Excellence (NICE), which is based on the administration of thiamine IV at a dose of 500 mg every eight hours for five days, combined with intravenous multivitamins, with correction of hypomagnesemia and hydro electrolytic disorders. The glucose-containing solutes are to be avoided at the beginning of the treatment. Oral relays for at least three months can be indicated [11, 15].

The evolution is marked by the disappearance of clinical signs and MRI images if treatment is started early. However, in the event of a delay in diagnosis or treatment, the progression is towards neurological sequelae such as Korsakoff’s syndrome (atrophy of the mammary bodies) or even death in 17% of cases [5, 11, 12].

To prevent malnourished patients or those receiving long-term parenteral nutrition from Gayet Wernicke’s encephalopathy, systematic supplementation with vitamin B1 (Thiamine) is crucial because not all parenteral nutritious solutions are enriched in thiamine [16].

## Conclusions

Gayet-Wernicke’s encephalopathy is a pathology that remains lethal in the absence of diagnosis and requires urgent treatment. The prevention of this entity requires the systematic supplementation of prolonged parenteral nutrition solutions not enriched with B vitamins in predisposed patients, and the correction of possible magnesium deficiency, an essential factor in the absorption of thiamine.

## Data Availability

The datasets used during the current case report are available from the corresponding author on reasonable request.

## Abbreviations

GWE: Gayet-Wernicke’s Encephalopathy
BMI: Body Mass Index
PS: Performance Statuts
GCS: Glasgow Coma Scale
CBC: Complete Blood Count
WBC: White Blood Cells
RBC: Red Blood Cells
Hb: Hemoglobin
HCT: Hematocrit
MCV: Mean Cell Volume
MCH: Mean Cell Hemoglobin
MCHC: Mean Cell Hemoglobin Concentration
RDW: Red Blood Cell Distribution Width
MPV: Mean Platelet Volume
Na^+^: Sodium
K^+^: Potassium
CT: Computed Tomography
MRI: Magnetic Resonance Imaging
NICE: National Institute of Health and Clinical Excellence
IV: Intravenous
